# Efficiency improvement of GaN-based ultraviolet light-emitting diodes with reactive plasma deposited AlN nucleation layer on patterned sapphire substrate

**DOI:** 10.1186/1556-276X-9-505

**Published:** 2014-09-16

**Authors:** Chia-Yu Lee, An-Jye Tzou, Bing-Cheng Lin, Yu-Pin Lan, Ching-Hsueh Chiu, Gou-Chung Chi, Chi-Hsiang Chen, Hao-Chung Kuo, Ray-Ming Lin, Chun-Yen Chang

**Affiliations:** 1 Department of Photonics and Institute of Electro-Optical Engineering, National Chiao Tung University, Hsin-Chu 30010, Taiwan; 2 Department of Electrophysics, National Chiao Tung University, Hsin-Chu 30010, Taiwan; 3 Microelectronic and Information System Research Center, National Chiao Tung University, Hsin-Chu 30010, Taiwan; 4 Advanced Optoelectronic Technology Incorporation, Hsinchu County 303, Taiwan; 5 Optorun Co., Ltd., Saitama-ken 350-0801, Japan; 6 Department of Electronic Engineering, Chang-Gung University, Taoyuan 333, Taiwan

**Keywords:** GaN, Flip chip ultraviolet light-emitting diodes (FC UV-LEDs), Nucleation, Reactive plasma deposited AlN

## Abstract

The flip chip ultraviolet light-emitting diodes (FC UV-LEDs) with a wavelength of 365 nm are developed with the *ex situ* reactive plasma deposited (RPD) AlN nucleation layer on patterned sapphire substrate (PSS) by an atmospheric pressure metal-organic chemical vapor deposition (AP MOCVD). The *ex situ* RPD AlN nucleation layer can significantly reduce dislocation density and thus improve the crystal quality of the GaN epitaxial layers. Utilizing high-resolution X-ray diffraction, the full width at half maximum of the rocking curve shows that the crystalline quality of the epitaxial layer with the (RPD) AlN nucleation layer is better than that with the low-temperature GaN (LT-GaN) nucleation layer. The threading dislocation density (TDD) is estimated by transmission electron microscopy (TEM), which shows the reduction from 6.8 × 10^7^ cm^−2^ to 2.6 × 10^7^ cm^−2^. Furthermore, the light output power (LOP) of the LEDs with the RPD AlN nucleation layer has been improved up to 30 % at a forward current of 350 mA compared to that of the LEDs grown on PSS with conventional LT-GaN nucleation layer.

## Background

The emission wavelength of GaN-based semiconductor, a directly transitional wide bandgap material, is theoretically capable of covering the whole visible spectrum from UV to IR, and GaN-based semiconductors attract considerable attention due to their continuously expanding applications for optoelectronic devices, such as light emitting diodes (LEDs) and laser diodes (LDs) [1,2]. Recently, the applications of UV-LEDs with emission wavelengths of about 365 nm are widely expanding, such as in sterilization, medicine, biochemistry, water purification system, light sources for optical recording, fluorescence analyzer, biological sensor, and air purification systems. However, the external quantum efficiency (*η*_ex_) of UV-LEDs is still much lower than blue LEDs, including the differences between LED structural design, chip area, or other package design. Yamada et al. reported that *η*_ex_ was improved up to 35 % by using patterned sapphire substrate (PSS) [3]. The enhanced light extraction efficiency by scattering the emission light in the epi-layers has been considered, and also related reports demonstrate that the crystal quality can be enhanced by using PSS [4-6]. Despite this, the performance of UV-LEDs is sensitive to defects in epitaxial layer because of the lack of localized states in the multiple-quantum-well (MQW) active regions [7,8]. Therefore, improvement of GaN crystal quality for UV-LED is a crucial issue in order to promote related applications. A nucleation layer of GaN hetero-epitaxially grown on PSS is the most important factor for suppressing the formation of threading dislocation densities (TDDs). Lai et al. [9] have recently reported that the *ex situ* sputtered AlN nucleation layer prepared by radio-frequency (RF) sputtering could reduce the TDDs of GaN and enhance the LED performances due to improvement on crystal quality. The surface of PSS could be damaged by recoil argon ions, though, owing to higher bias voltage (200 ~ 400 V) of RF sputtering system and a short distance from the target to the sample. Thus, it is necessary to deposit AlN nucleation layer on PSS but not cause PSS surface damages. In this study, we demonstrated an UV-LEDs with an *ex situ* reactive plasma deposited (RPD) AlN nucleation layer on PSS. Comparing the RF sputtering system, the RPD system utilizes a lower bias voltage (15 ~ 20 V), and the distance between the target and the sample is longer. It is practical for avoiding the substrate from being damaged. Moreover, the deposition temperature of RPD AlN nucleation layer was kept at high temperature (600°C) that could lead to the preferred orientation growth. Systematic experiments and investigations have been described in detail, which showed an up to 30 % output performance increase by using RPD AlN nucleation layer on PSS.

## Methods

All samples were grown on 2-in. PSS by an AP-MOCVD system. The PSS was prepared using a cone pattern on the (0001) sapphire, which was fabricated by inductively coupled plasma reactive ion etching in order to etch (0001) the sapphire-coated cone-shaped photoresistant layer. The bottom diameter, the center-to-center spacing, and the height of the PSS were 2.5, 3, and 1.5 μm, respectively. After preparing the patterned substrates, a 25-nm-thick RPD AlN nucleation layer was deposited onto the PSS by Optorun RPD system (Optorun Co., Ltd., Saitama, Japan).

During an epitaxial process, trimethylgallium (TMGa), trimethylaluminum (TMAl), trimethylindium (TMIn), and ammonia (NH_3_) were employed as the reactant source materials for Ga, Al, In, and N, respectively. Hydrogen and nitrogen were used as carrier gases, and silane and bis-cyclopentadienyl magnesium (Cp2Mg) were used as sources for n-type and p-type dopants, respectively.

**Figure 1:**
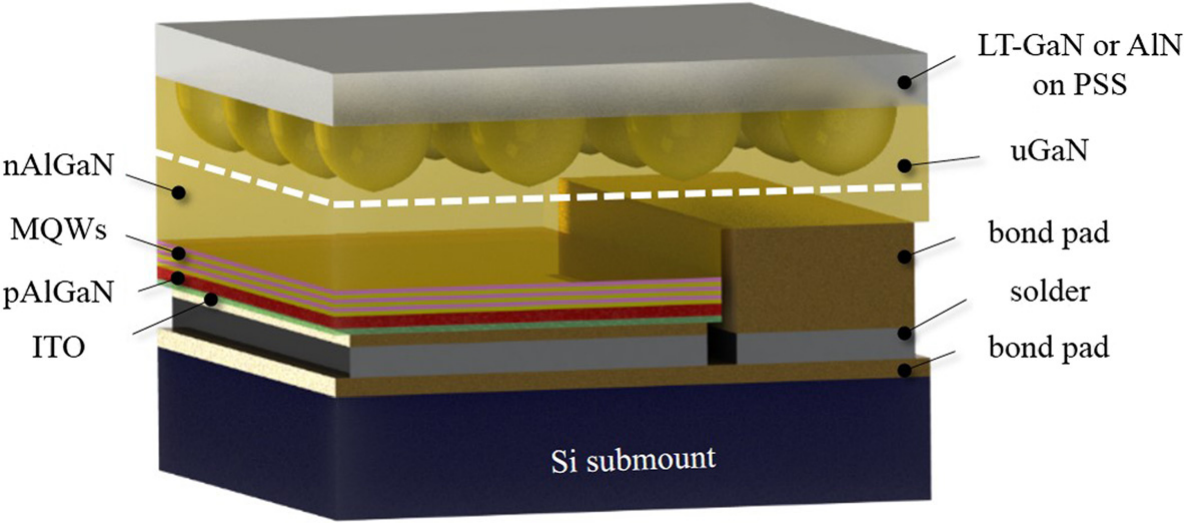
Schematic of the FC UV-LEDs with two kinds of nucleation layer.

Two samples were prepared: sample 1 was a device with a 3-μm-thick unintentionally doped GaN (u-GaN) layer which was grown on PSS using RPD AlN nucleation layer at 1,150°C where ELOG method was applied for fully coalesced GaN layer and the RPD AlN nucleation layer without thermal annealing treatment. By contrast, sample 2 has a 25-nm-thick low-temperature GaN (LT-GaN) nucleation layer, grown on PSS at a temperature of 520°C with thermal annealing treatment before the u-GaN epitaxial layer at 1,150°C. Following, the GaN-based LED structures were grown on both samples identically; the LED structures consisted of a 2.5-μm-thick n-type Al_0.02_Ga_0.98_N layer (n-doping is 5 × 10^18^ cm^−3^) with a temperature of 1,150°C, ten pairs of InGaN/InAlGaN MQWs with a 2.5-nm-thick un-doped well and a 12.5-nm-thick Si-doped barrier as active layers grown at 830°C, a 15-nm-thick Mg-doped Al_0.3_Ga_0.7_N and a 10-nm-thick Mg-doped Al_0.1_Ga_0.9_N electron blocking layers (EBL) grown at 1,050°C (p-doping = 1 × 10^17^ cm^−3^), a 50-nm-thick Mg-doped GaN cap layer (p-doping = 5 × 10^17^ cm^−3^) grown at 1,030°C, and a 4-nm-thick p-type InGaN contact layer. The crystalline qualities of these LED samples with RPD AlN nucleation layer (i.e., LED I) and LT-GaN nucleation layer (i.e., LED II) were then investigated by performing high-resolution X-ray diffraction (HRXRD) and transmission electron microscopy (TEM). Subsequently, the LED mesa with a pattern of 45 × 45 mil^2^ was defined and fabricated by photolithography and dry etching. A transparent conduction indium tin oxide (ITO) layer was employed to be a p-type ohmic contact layer. Finally, a Ni/Ag/Pt and Ti/Pt/Au metallization was deposited as p-type and n-type electrodes, respectively. After conventional LED processes, flip chip technology was applied for better light extraction and heat dissipation [10]. The LED chips with patterned sapphire substrate were flip chip bonded onto silicon submount by using Panasonic ultrasonic flip chip bonder (Panasonic, Kadoma, Osaka, Japan). Figure 1 shows the schematic diagram of the finished flip chip ultraviolet light-emitting diodes (FC UV-LEDs). The light output power-current-voltage (*L*-*I*-*V*) characteristics of UV-LEDs were measured under CW operation by a conventional probe station and an integrated sphere instrument at room temperature.

## Results and discussion

**Figure 2:**
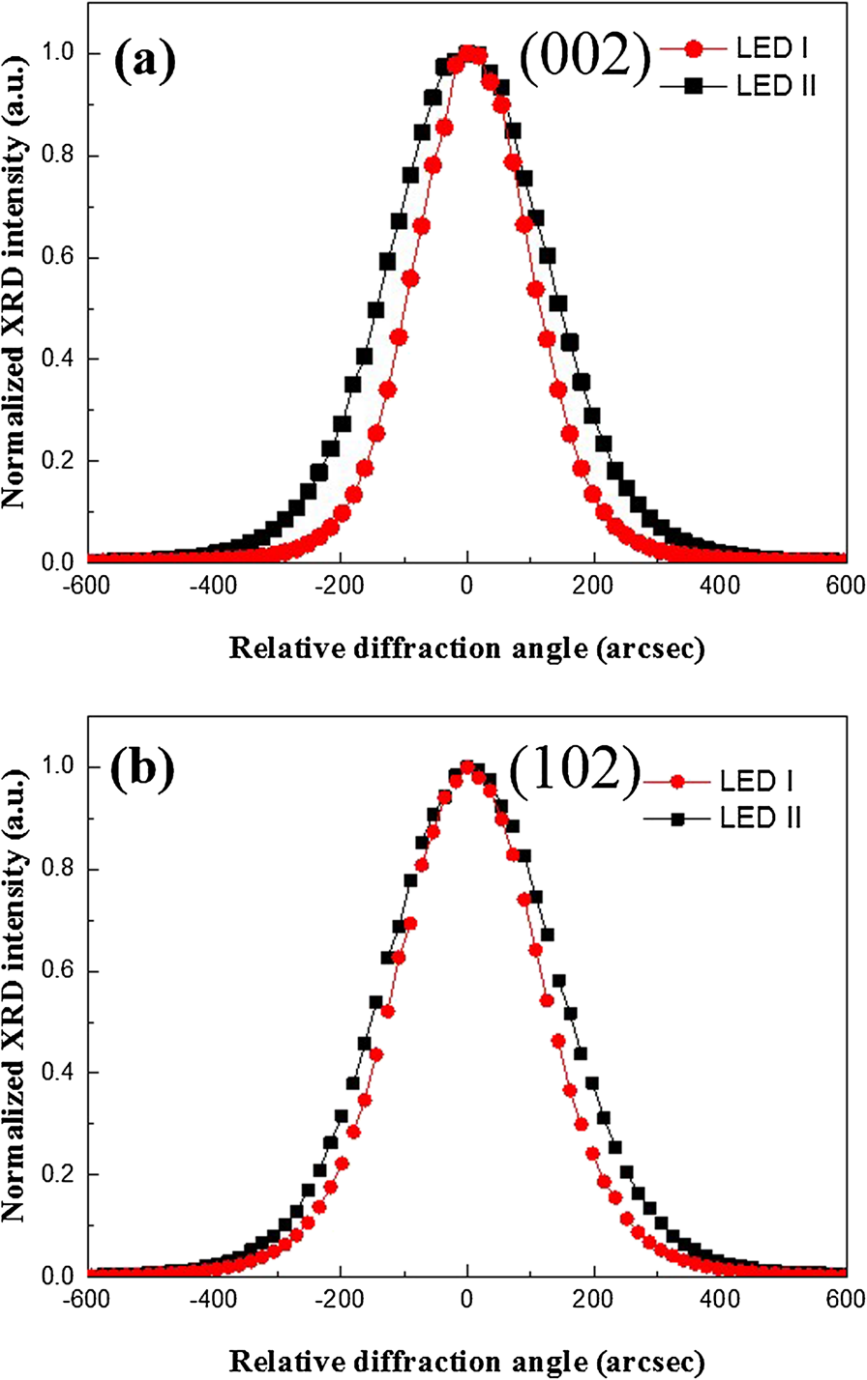
**HRXRD ω**-**rocking scans of (a) GaN (002) and (b) GaN (102) for GaN epitaxial layers.**

The n-GaN epitaxial films of both samples with the RPD AlN nucleation layer and with the LT-GaN nucleation layer are characterized by triple-axis HRXRD. In wurtzite GaN films, the full width at half maximum (FWHM) of the symmetric (002) and asymmetric (102) ω-scan spectra are affected by edge and screw and/or mixed dislocations [11]. As shown in Figure 2, the fitted FWHM of (002) and (102) rocking curve is reduced from 291 to 218 arc sec and from 320 to 263 arc sec, respectively. The result shows clear evidence that GaN crystalline quality grown on PSS with RPD AlN nucleation layer is better than that grown on PSS with LT-GaN nucleation layer.

**Figure 3:**
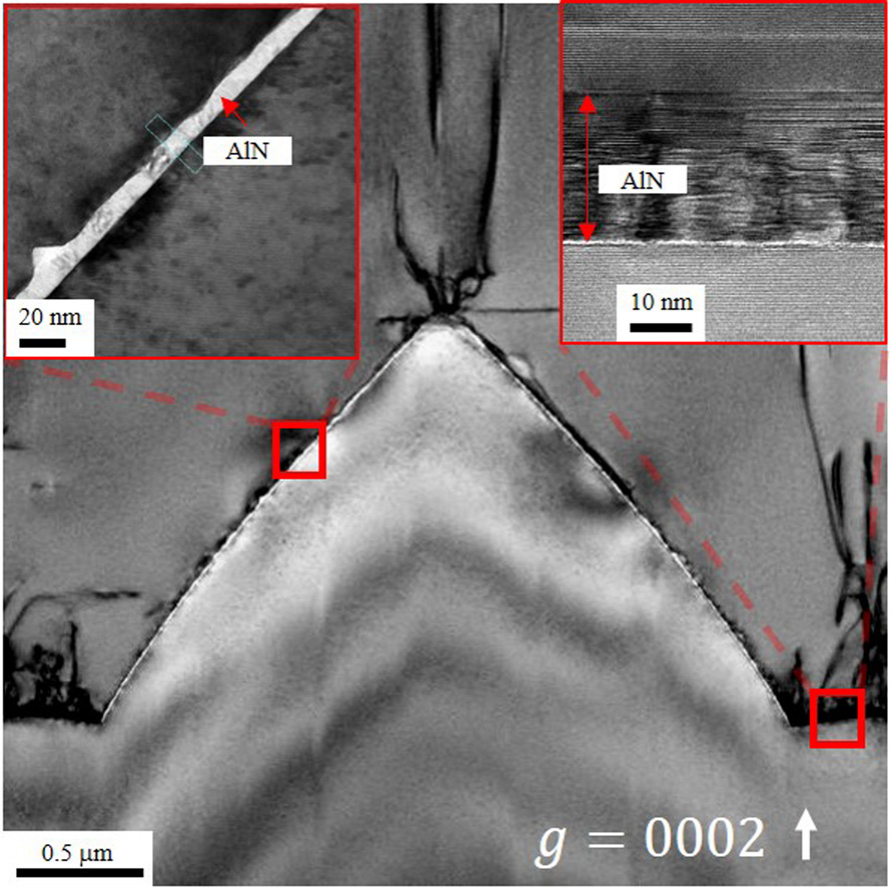
Cross-sectional TEM images of GaN with RPD AlN nucleation layer.

**Figure 4:**
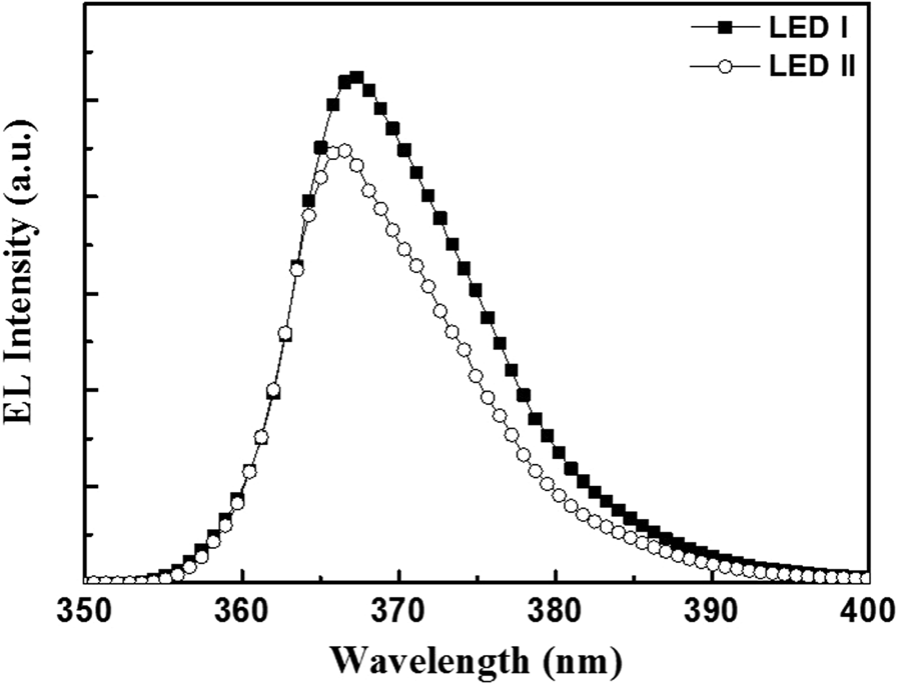
The EL spectra of the LED devices measured at 350 mA.

In order to investigate the quality of u-GaN on PSS, TEM measurement was used. Figure 3 shows a resultant TEM cross-section image of u-GaN grown on PSS with RPD nucleation layer, in which an extremely thin AlN layer uniformly covering on PSS was observed. Two insets of Figure 3 are magnification images of the AlN nucleation layer denoted by the red square: on the right shows the magnified image at the bottom of PSS and on the left displays the magnified image at the side wall of PSS. From the TEM image of AlN located at the bottom of PSS, a mixed crystallized and amorphous AlN of 25 nm on sapphire was observed, and from the TEM image of AlN position at the side wall of PSS, a 9-nm-thick AlN nucleation layer with excellent uniformity can be seen. The inconsistency of thickness of AlN between the bottom and side wall of PSS might be the result of the morphology and crystal orientation. It is noteworthy that no GaN islands appear on the surface of PSS. Generally, with the conventional epitaxial growth of GaN with LT-GaN nucleation layer, the GaN islands are formed during the initial steps of epitaxy and subsequently expand to coalesce with each other during the formation of GaN thin film. The threading dislocations are generated at the boundary of those GaN islands. The nucleation of GaN islands conducts on various crystalline planes of sapphire, rather than single c-plane sapphire, which results in a higher concentration of defects due to lattice mismatch between the sapphire and c-plane GaN on the side wall of PSS [12]. Similarly, the GaN islands which appear at the bottom of PSS initially, a single c-plane GaN could be grown on c-plane sapphire owing to the lower concentration of defects. Although the quality of GaN islands can be improved, threading dislocations are formed at the boundary between GaN islands during coalescence of the islands [13]. The elimination of those unfavorable GaN islands is achieved by the AlN nucleation layer as what was observed in the TEM image. Therefore, the AlN nucleation layer can effectively suppress the formation of threading dislocations from appearing in GaN islands. In addition, the edge-type dislocation is estimated from 6.8 × 10^7^ cm^−2^ to 2.6 × 10^7^ cm^−2^ by TEM two-beam condition analysis [14]. The results consist of the observations in XRD ω-scan spectra shown in Figure 2.The emission wavelengths of the fabricated LEDs were examined by electroluminescence (EL) measurements under room temperature at a driving current of 350 mA. Both the LEDs grown on PSS with RPD AlN nucleation layer and with LT-GaN nucleation layer display peak wavelength of emission at 365 nm, as shown in Figure 4. Furthermore, the EL peak intensity of the LED grown on PSS with RPD AlN nucleation layer is stronger than that with LT-GaN nucleation layer, reflecting the defect density of the LED grown on PSS with RPD AlN nucleation layer, which is much lower than that with LT-GaN nucleation layer. The enhancement of EL intensity observed on the LED grown on PSS with RPD AlN nucleation layer is dominated by the lower concentration of defects, since the LED structures of the two samples are similar.

**Figure 5:**
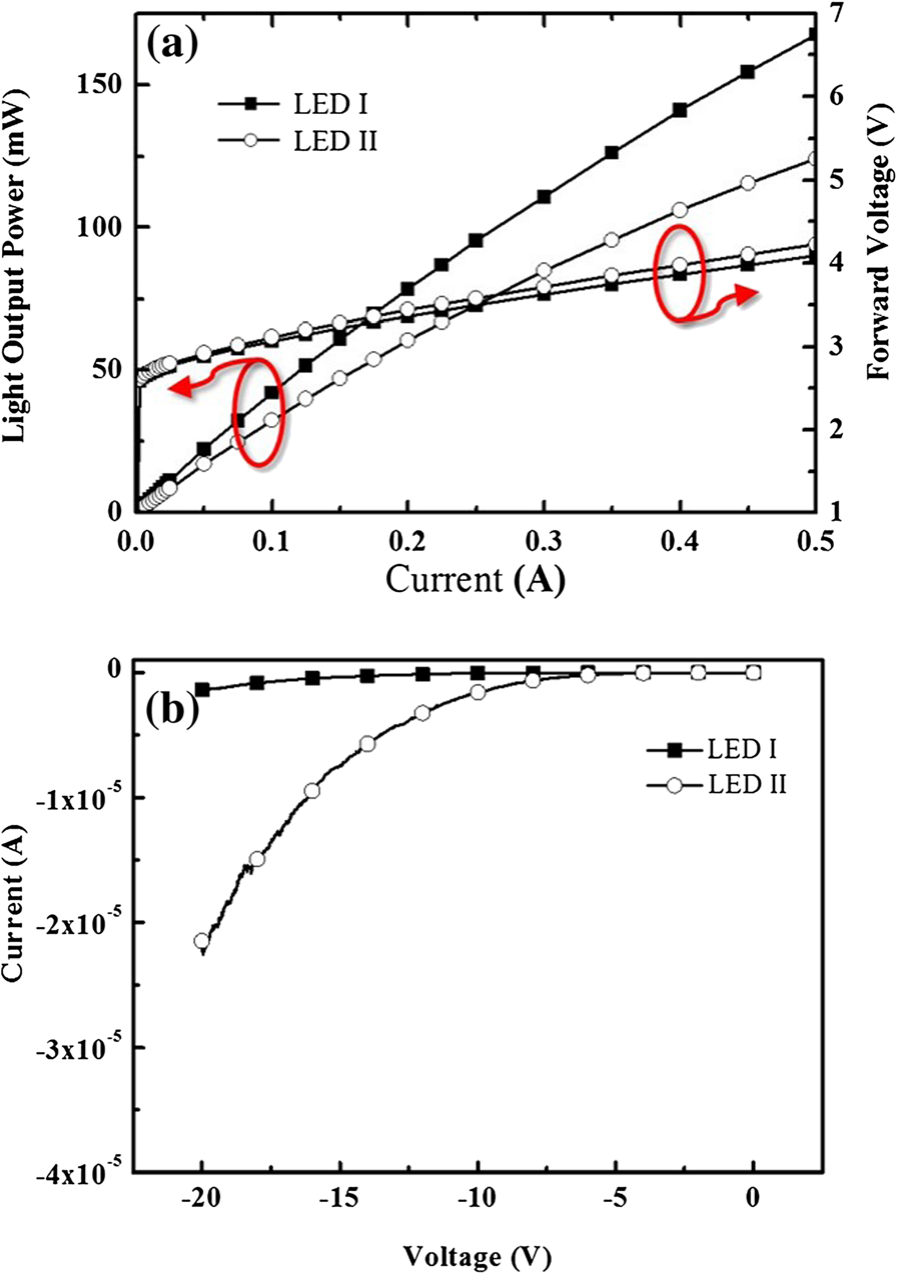
**The (a) *L*- *I *-*V *** **characteristics and the (b) reverse leakage currents of fabricated LED devices.**

The light output versus driving current and voltage (*L*-*I*-*V*) measurements were conducted on the two LEDs to investigate the device performance at room temperature. The results are shown in Figure 5a, where a current injection is 350 mA. It displays that the forward voltages are 3.59 and 3.78 V, while the light output powers (LOP) are 126 and 95 mW for LED I and LED II, respectively. The 350-mA LOP of LED I (with the RPD AlN nucleation layer) is enhanced by 32.6 % compared to that of LED II (with the conventional MOCVD-grown LT-GaN nucleation layer). The improvement of LOP shows that the internal quantum efficiency (IQE) of LED I is higher than that of LED II, as previously stated, where the optical characteristics affected by the structure of two devices are similar. Given that external quantum efficiency (EQE) is the product of light extraction efficiency (LEE) and IQE, a higher EQE is the result of a higher IQE with similar LEE. Here, LED I and LED II have the same geometric structure; therefore, the LEE can be considered similar. The LOP improvement of LED I is attributed to increasing IQE, which is associated with the improved crystal quality of the device. The reverse leakage currents of LED I and LED II are shown in Figure 5b. At a reverse bias of −20 V, LEDs I and II show the current leakage of −1.37 and −21.51 μA, respectively. The reverse current leakage of LED I is less than that of LED II by an order of reverse bias of −20 V. Since the current leakage of the LED device is principally influenced by threading dislocation, the crystalline quality of GaN is the key. Consequently, the modification of current leakage of LED I with the RPD AlN nucleation layer is a direct result of crystalline quality improvement of GaN.

**Figure 6:**
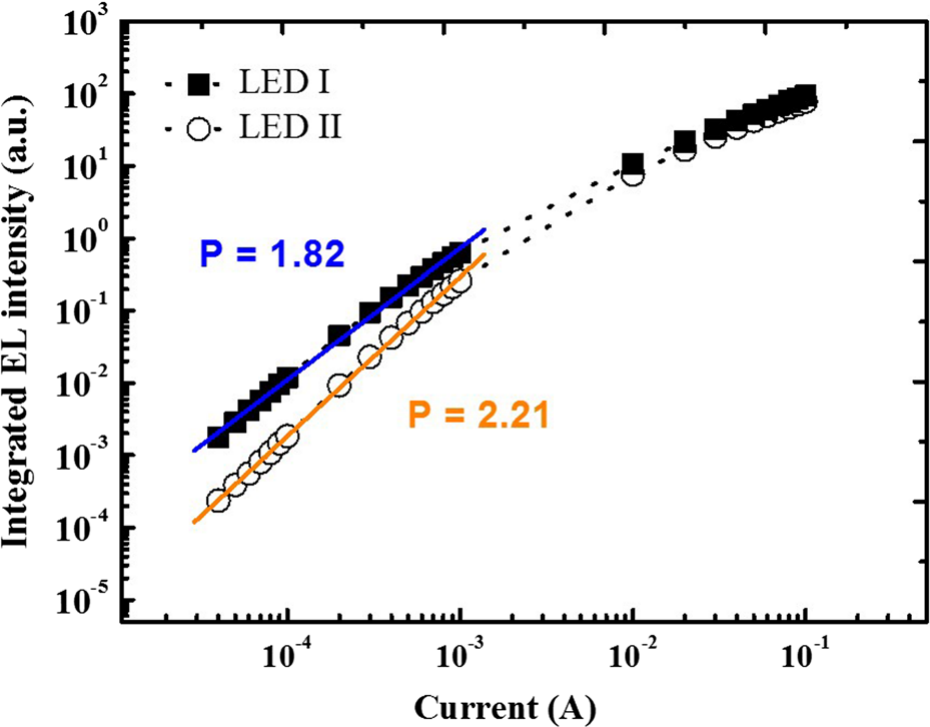
Power-dependent electroluminescence (PD-EL) measurements of two LED devices.

A power-dependent electroluminescence (PD-EL) measurement of LED I and LED II was conducted under room temperature for comparison. The results are shown in Figure 6. From the EL spectra, it shows that the EL intensity of LED I is thoroughly stronger than that of LED II at all driving currents, indicating less non-radiative recombination centers in LED I. The inference is further confirmed by the power law of *L*-*I* characteristics:

(1) $$L\propto I^{P}\text{,}$$

where *L* is the EL luminescence, *I* is the driving current, and *P* is a constant indicating the contribution of non-radiative recombination to overall recombination balance. A higher value of *P* constant indicates a higher concentration of non-radiative recombination centers. At the linear region of the power law fitting curve of PD-EL, the obtained *P* constants for LED I and LED II are 1.82 and 2.21, respectively; non-radiative recombination centers are greatly decreased in LED I. With the driving current increasing, the increasing rate of PD-EL curves of two devices decrease and gradually merge with each other as one. It is dominated by non-radiative Auger recombination, since the driving current in this region is high. The two LEDs are quenched at high driving current, so that the two curves are gradually becoming identical. LED I and LED II have similar geometric structures, except for the nucleation layer which causes different GaN growth crystal quality, that influence performance of the devices.

## Conclusions

In this study, a RPD AlN nucleation layer has been utilized to improve the crystal quality of GaN on PSS. The better crystallinity of GaN with RPD AlN nucleation layer compared to that with LT-GaN nucleation layer is confirmed by the XRD spectra. The TEM images show that the RPD AlN nucleation layer possesses good coverage uniformity and effectively suppresses the formation of threading dislocations by eliminating GaN islands on PSS. The room temperature EL spectra of the LED with RPD AlN nucleation layer show stronger luminescence intensity compared to that of conventional LEDs. The LOP of the LED with the RPD AlN nucleation layer is enhanced by 32.6 % compared to that of the LED with the conventional LT-GaN nucleation layer at 350 mA. Both the observations of *L*-*I*-*V* curves and current leakage at reverse bias indicate the improvement of crystal quality brought by AlN nucleation layer. PD-EL measurement has also been conducted on the two LEDs for further confirmation, indicating that less non-radiative recombination centers are performed in the LED with AlN nucleation layer. All observations and analysis have consistently shown that the AlN nucleation layer can significantly improve the performance of a LED by increasing the crystal quality of GaN.

## Competing interests

The authors declare that they have no competing interests.

## Authors’ contributions

C-YL carried out the experimental work including all the measurements. C-YL and A-JT drafted the manuscript. Ci-HC prepared the RPD AlN nucleation layer on PSS. B-CL and Cg-HC achieved the fabrication of LED devices. Y-PL conducted the experimental design and result analysis. R-ML and H-CK provided suggestions and comments on epitaxial growth. G-CC and C-YC participated in all the discussions on this study. All authors read and approved the final manuscript.
